# AF10 (*MLLT10*) prevents somatic cell reprogramming through regulation of DOT1L-mediated H3K79 methylation

**DOI:** 10.1186/s13072-021-00406-7

**Published:** 2021-07-02

**Authors:** Deniz Uğurlu-Çimen, Deniz Odluyurt, Kenan Sevinç, Nazlı Ezgi Özkan-Küçük, Burcu Özçimen, Deniz Demirtaş, Eray Enüstün, Can Aztekin, Martin Philpott, Udo Oppermann, Nurhan Özlü, Tamer T. Önder

**Affiliations:** 1 School of Medicine, Koc University, Istanbul, 34450 Turkey; 2 Department of Molecular Biology and Genetics, Koc University, Istanbul, 34450 Turkey; 3 Botnar Research Centre, University of Oxford, Oxford, UK; 4 Centre for Medicine Discovery, University of Oxford, Oxford, UK

**Keywords:** AF10, DOT1L, BioID, Reprogramming, iPSC, H3K79 methylation

## Abstract

**Background:**

The histone H3 lysine 79 (H3K79) methyltransferase DOT1L is a key chromatin-based barrier to somatic cell reprogramming. However, the mechanisms by which DOT1L safeguards cell identity and somatic-specific transcriptional programs remain unknown.

**Results:**

We employed a proteomic approach using proximity-based labeling to identify DOT1L-interacting proteins and investigated their effects on reprogramming. Among DOT1L interactors, suppression of AF10 (*MLLT10*) via RNA interference or CRISPR/Cas9, significantly increases reprogramming efficiency. In somatic cells and induced pluripotent stem cells (iPSCs) higher order H3K79 methylation is dependent on AF10 expression. In AF10 knock-out cells, re-expression wild-type AF10, but not a DOT1L binding-impaired mutant, rescues overall H3K79 methylation and reduces reprogramming efficiency. Transcriptomic analyses during reprogramming show that AF10 suppression results in downregulation of fibroblast-specific genes and accelerates the activation of pluripotency-associated genes.

**Conclusions:**

Our findings establish AF10 as a novel barrier to reprogramming by regulating H3K79 methylation and thereby sheds light on the mechanism by which cell identity is maintained in somatic cells.

**Supplementary Information:**

The online version contains supplementary material available at 10.1186/s13072-021-00406-7.

## Background

The low efficiency of transcription factor-based reprogramming points to the presence of multiple rate-limiting steps or barriers to cell fate changes [1]. We have previously identified the histone H3 Lysine 79 (H3K79) methyltransferase DOT1L as one of the key barriers to reprogramming of somatic cells to pluripotency [2]. DOTL1 inhibition can functionally replace KLF4 and c-MYC [2], increase reprogramming efficiency in a wide range of systems [3–6], facilitate the generation of chemically induced pluripotent stem cells (ciPSCs) from mouse somatic cells [7] and result in a permissive epigenome state which enables reprogramming by alternative transcription factors [8]. DOT1L is recruited to RNAPII-associated transcription-elongation machinery through a number of interacting proteins that include members of AEP (AF4 family/ENL family/P-TEFb), EAP (ENL-associated proteins), DotCom, and super-elongation protein complexes [9–12]. H3K79 methylation decorates actively transcribed gene bodies where it can act as an anti-silencing mark and prevent the recruitment of repressive chromatin modifiers [13–16]. In the context of reprogramming, DOT1L activity serves to maintain the expression of somatic-specific genes and prevents mesenchymal-to-epithelial transition (MET), an important step in the process [2]. However, the key interaction partners of DOT1L which play a role in safeguarding somatic cell identity remain unknown. In the present work, we addressed this question using a combination of proteomics and loss of function approaches and identified AF10 as a key DOT1L-interacting protein in maintaining cell identity.

## Results

### Identification of proximal interactors of DOT1L via BioID

**Fig. 1:**
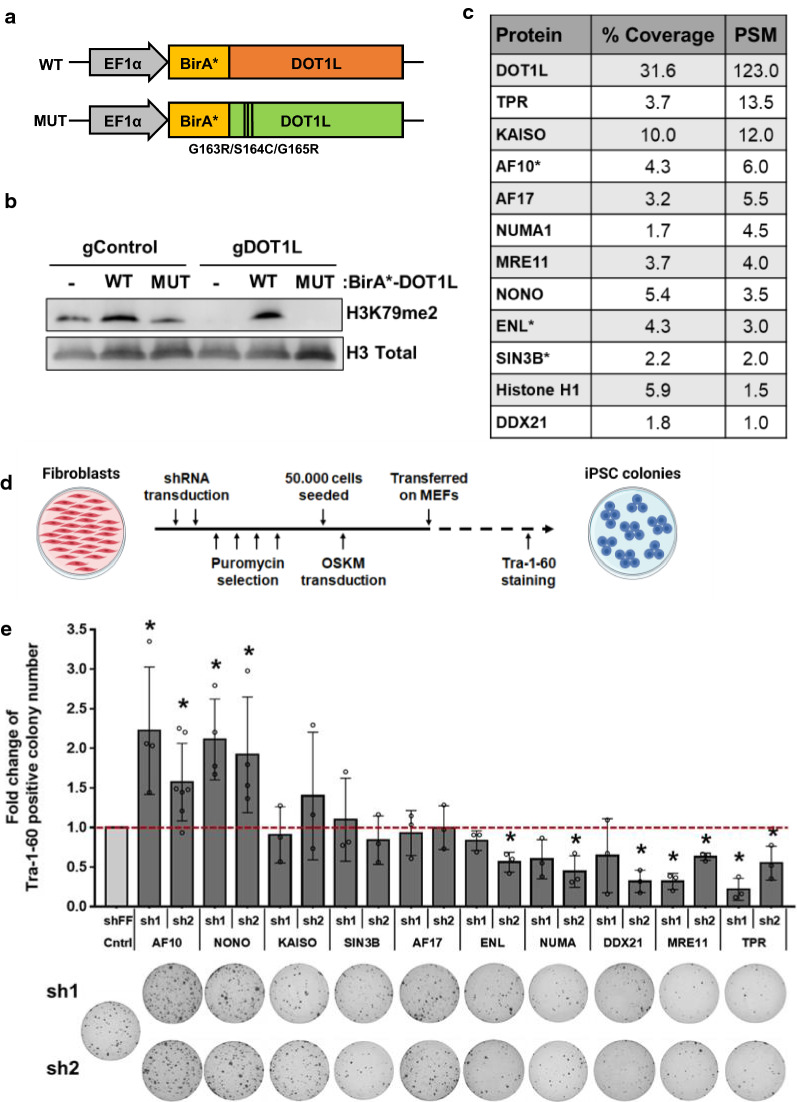
Identification of proximal interactors of DOT1L via BioID. **a** Schematic of BirA*-DOT1L fusion protein-expressing vector constructs. **b** H3K79 di-methylation (H3K79me2) levels in control (gControl) and DOT1L knock-out (gDOT1L) cells expressing either WT or MUT BirA*-DOT1L fusion constructs. Total histone H3 was used as a loading control. **c** Proximal-protein interactions of DOT1L as revealed via proteomic analyses of biotinylated proteins. Proteins were ordered according to their average coverage and PSM (peptide spectrum matches) values in BirA*-DOT1L wt expressing cells. Asterisk indicates proteins detected only in wt-DOT1L samples. **d** Schematic of shRNA-mediated somatic cell reprogramming timeline. **e** Bar graph represents fold change in reprogramming efficiency upon shRNA-mediated gene silencing. Tra-1-60 positive colony numbers of each experiment were normalized to shControl sample. Average of fold changes from independent experiments are indicated (circles). Representative Tra-1-60 stained well images for each shRNA-infected sample are displayed under the bar graph. Error bars represent SEM. *P < 0.05

To identify interaction partners of DOT1L in somatic cells, we generated a fusion protein linking a promiscuous biotin ligase (BirA*) with DOT1L (Fig. 1a) [17]. We also generated a BirA*-fusion with a catalytically dead DOT1L mutant (G163R/S164C/G165R) incapable of H3K79 methylation to assess if putative interactors could be dependent on catalytic activity of DOT1L (Fig. 1a) [18]. To test the functionality of these fusion proteins, constructs were transfected into control and DOT1L knock-out  HEK293T cells generated via CRISPR/Cas9. In the DOT1L knock-out background (guideRNA DOT1L-gDOT1L), H3K79 methylation was restored upon expression of wild-type, but not mutant DOT1L fusion protein, confirming that BirA*-fusion does not interfere with catalytic activity (Fig. 1b, Additional file 1: Fig. S1a). Biotinylated proteins were enriched with Streptavidin pulldown and analyzed in LC–MS/MS. Mass spectrometry analysis resulted in detection of DOT1L with the highest PSM (peptide spectrum matches) values (1% false discovery rate (FDR)) and high sequence coverage (30%) in fusion protein-expressing samples; whereas none was detected in control samples as expected. In wt-DOT1L fusion expressing samples, 11 proteins were identified (Fig. 1c). Among these were a number of previously characterized interactors such as AF10 (*MLLT10*), AF17, ENL as well six novel putative proximal-interactor proteins (TPR, KAISO, NUMA1, MRE11, NONO, SIN3B). Analysis of the identified hits with the Contaminant Repository for Affinity Purification (CRAPome) database showed that among putative DOT1L-interactors, AF10, SIN3B and KAISO is highly specific to BioID assay (Additional file 1: Figure S1b) [19]. In contrast, 106 proximal interactors were detected in mut-DOT1L expressing cells (Additional file 2: Table S1). This larger number of biotinylated proteins in mut-DOT1L samples may be due to a defect in chromatin localization of the mutant protein, a notion that needs further investigation. Among putative interactors of DOT1L, only three proteins (AF10, ENL and SIN3B) were specific to wt-DOT1L (Fig. 1c).

We next asked whether any of the putative interactors of wt-DOT1L have an effect on the reprogramming of human fibroblast to iPSCs. For reprogramming experiments, human embryonic stem cell H1-derived fibroblasts (dH1f) were used [20]. In a loss of function approach, we knocked-down individual candidate genes by two independent shRNAs. The majority of shRNAs achieved at least 50% knock-down of their respective target gene (Additional file 1: Figure S1c). Reprogramming was initiated after shRNA transduction and the resulting iPSC colonies were identified via Tra-1-60 expression, a well-established marker of fully reprogrammed cells (Fig. 1d) [21]. We observed that knock-down of AF10 and NONO significantly increased the number of iPSC colonies, resulting in 1.5 to two fold greater reprogramming efficiency compared to control shRNA expression (Fig. 1e). On the other hand, knock-down of MRE11 and TPR decreased reprogramming significantly (Fig. 1e). We also tested the effect of suppressing AF9 in reprogramming. AF9 is a well-characterized DOT1L interactor, but was not identified as a hit in our MS analysis [22]. Inhibition of AF9 by two independent shRNAs had either no effect or led to a slight decrease in reprogramming efficiency (Additional file 1: Figure S1d, e).

### AF10 suppression enhances reprogramming

**Fig. 2:**
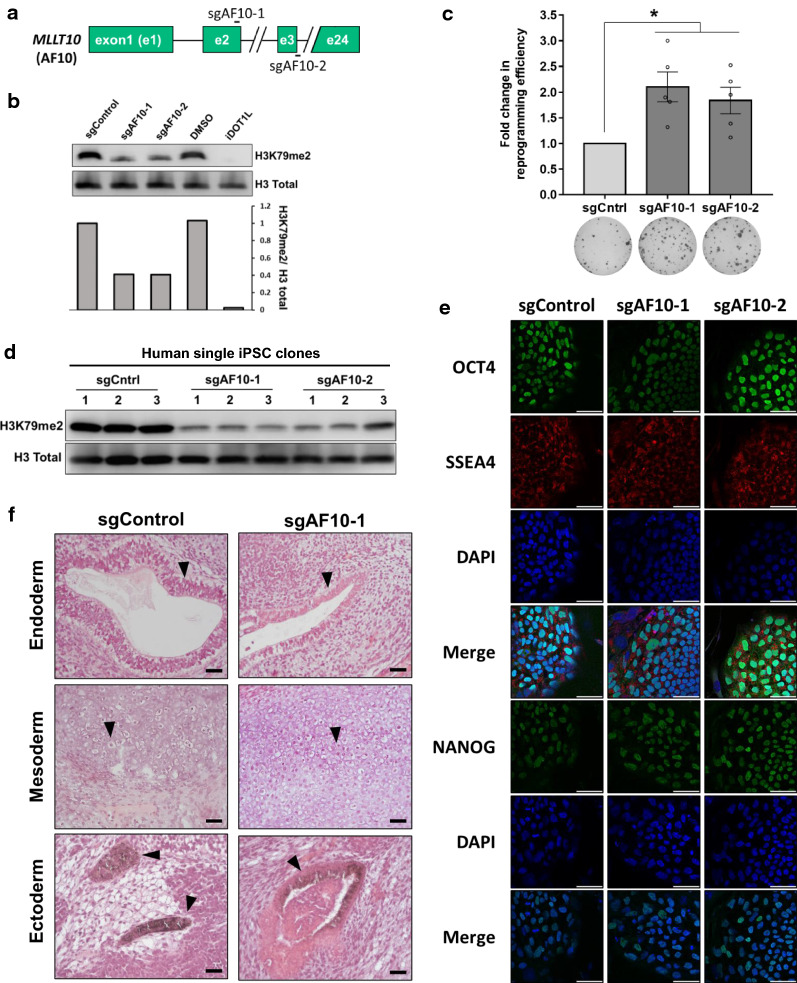
AF10 regulates H3K79 methylation and is a barrier to reprogramming. **a** Schematic for AF10 (*MLLT10)* gene indicating target sites for the AF10 sgRNAs. **b** H3K79me2 upon sgRNA-mediated AF10 knock-out. iDOT1L (EPZ004777) was used as a positive control of H3K79me2 depletion. Fibroblasts were treated with DMSO or 3 μM iDOT1L for 10 days. sgAF10 infected dH1fs were selected with puromycin and cultured for 1 week. Total H3 levels are used as loading control. Quantifications were normalized to sgControl sample. **c** Fold change in the number of Tra-1-60 positive colonies upon sgAF10 expression. *P* values were determined by one sample *t*-test; *P < 0.05. Bar graphs show the mean and error bars represent SEM in independent biological replicates (each circle). Representative Tra-1-60 stained wells are shown below the graph. *P* values were 0.009 for sgAF10-1 and 0.016 for sgAF10-2. **d** Immunoblot for H3K79me2 in single-cell clones of sgControl and sgAF10 iPSC lines. Total H3 levels were used as loading control. **e** OCT4, SSEA4 and NANOG immunofluorescence of iPSCs derived from sgControl and sgAF10 expressing fibroblasts. DAPI was used to stain the nuclei. Scale bars represent 50 μm. **f** Hematoxylin and eosin stained sections of teratomas generated by iPSCs derived from sgControl and sgAF10-1 cells. Black arrowheads show glandular epithelium (endoderm, top), cartilage tissue (mesoderm, middle), and pigmented neural tissue (ectoderm, bottom). Representative images are from one of two independent teratomas. Scale bars represent 20 μm

We were intrigued by the increased reprogramming efficiency upon AF10 and NONO knock-down and followed up on these two candidate genes. We next asked if these two proteins play a role in regulating cellular H3K79 methylation levels. Knock-down of *NONO* did not change total H3K79me2 levels (Additional file 1: Figure S1f). Considering that Nono has been shown to limit self-renewal of mESCs by regulating bivalent gene expression, reprogramming enhancement upon *NONO* knock-down may occur independent of H3K79 methylation [23]. In contrast, AF10 inhibition via shRNAs significantly decreased H3K79 methylation (Additional file 1: Figure S1f). To further confirm the role of AF10 in reprogramming, we pursued an independent strategy to inhibit AF10 using two independent single guide RNAs targeting splice site exon 2 (sgAF10-1) or exon 3 of *MLLT10* (sgAF10-2) [24] (Fig. 2a). CRISPR-targeted sites were verified via T7 endonuclease assay via cleavage of heteroduplex DNA fragments (Additional file 1: Figure S2a). In addition, sgAF10-expressing fibroblasts had lower *AF10* mRNA levels compared to sgControl-expressing cells (Additional file 1: Figure S2b). H3K79 methylation was decreased in both sgAF10 cell lines, albeit to a lesser degree than treatment with a small molecule inhibitor of DOT1L (iDOT1L, EPZ004777) (Fig. 2b, Additional file 1: Fig. S2c). sgAF10 expressing-fibroblasts generated up to twofold greater number of iPSC colonies compared to control cells (Fig. 2c). We next evaluated if iPSCs derived via AF10 suppression were bona fide pluripotent cells. AF10 and H3K79me2 levels were significantly reduced in the majority of sgAF10-derived iPSC single-cell clones tested (Fig. 2d, Additional file 1: Fig. S2d). sgAF10 iPSC colonies were positive for OCT4, SSEA4 and NANOG at the protein level, and, upon injection into immunodeficient mice, readily formed teratomas containing cells originating from all three germ layers (Fig. 2e, f). Teratoma formation latency was similar in control and AF10 inhibited lines and were comparable to DOT1L-inhibited iPSC clones we previously generated [2]. Overall, these experiments show that cells with AF10 inhibition can be fully reprogrammed into bona fide iPSCs.

**Fig. 3:**
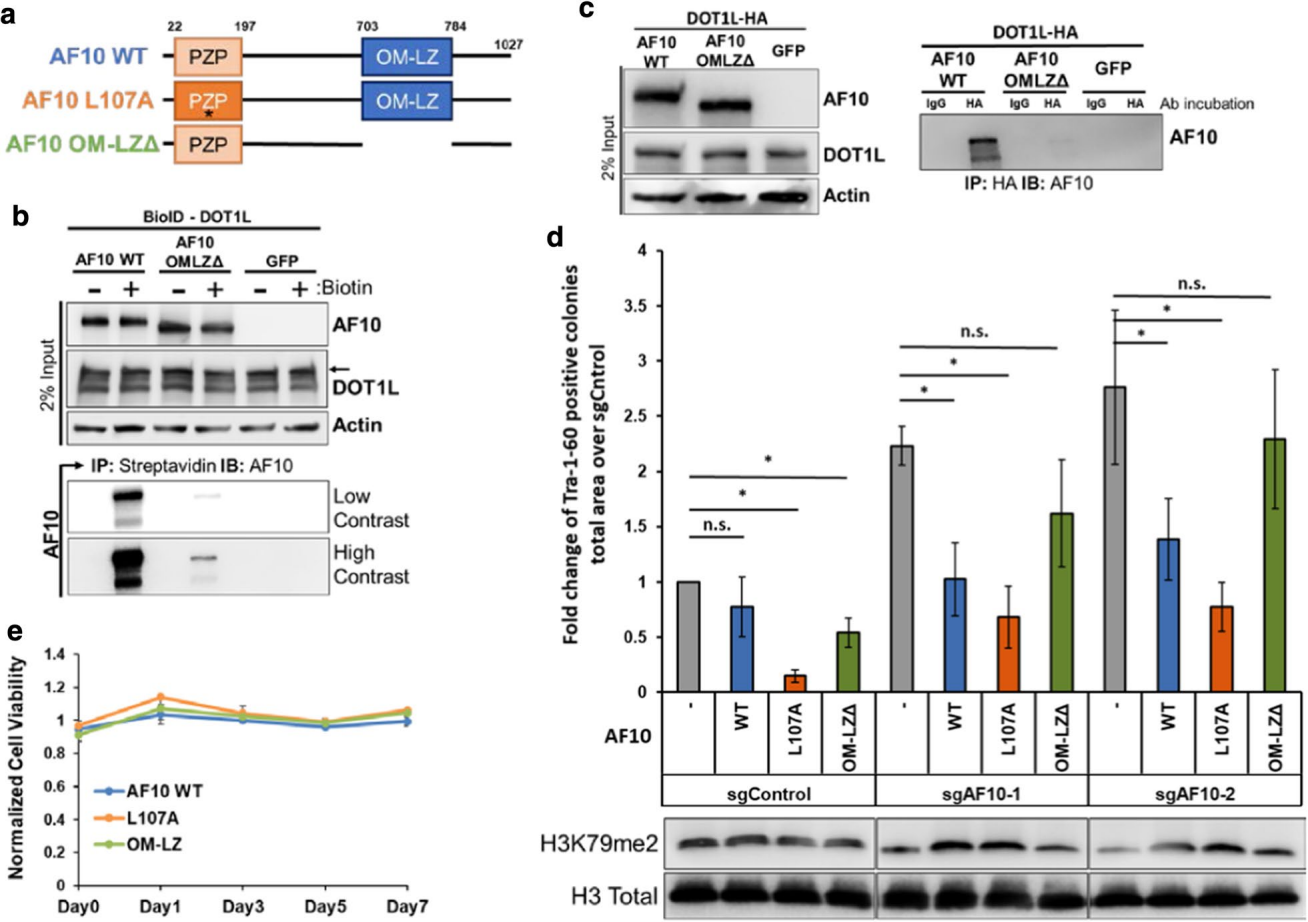
AF10 prevents reprograming through its interaction with DOT1L. **a** Domain organization of wild type and mutant AF10s used in **d**. L107A mutation abolishes Histone H3K27 binding and OM-LZ deletion impairs DOT1L binding. **b** Immunoblots for AF10 following streptavidin pulldown from cells expressing BioID-DOT1L and GFP, WT-AF10 or OM-LZ∆ AF10. Top panels show 2% of input samples and bottom panels show Streptavidin pull-down samples. (+) Biotin cells were treated with 50 μM D-Biotin 24 h. **c** Immunoblots for AF10 following immunoprecipitation with IgG or HA antibodies form cells expressing DOT1L-HA and GFP, WT-AF10 or OM-LZ∆ AF10. Left panels show 2% of input samples and right panel shows HA pull-down samples. **d** Fold change in the number of Tra-1-60-positive colonies derived from control or AF10 knock-out cells expressing WT, L107A or OM-LZ∆ AF10 cDNAs. *P* values were determined by one sample *t* test; *P < 0.05. Bar graph shows the mean and error bars represent SEM in 3 independent biological replicates. Bottom panel shows the H3K79me2 levels with H3 total as a loading control. **e** Relative cell viability of dH1f cells expressing WT, L107A or OM-LZ∆ AF10. Cell Titer Glo Assay measurements were normalized to uninfected dH1f cells across the indicated time-points. Error bars indicate standard deviation of triplicate samples

We next asked whether the increased reprogramming phenotype upon AF10 knock-out could be rescued by re-expression of AF10 (Fig. 3a). Wild-type AF10 cDNA increased overall H3K79me2 levels in sgAF10-expressing cells, and importantly, decreased the reprogramming efficiency (Fig. 3d, Additional file 1: Fig. S2e). Thus, the increased reprogramming phenotype upon AF10 silencing could be rescued by overexpression of WT-AF10. Using the same approach, we next asked if a H3K27 binding-mutant of AF10 (L107A) and a DOT1L-binding domain deleted AF10 (octapeptide motif-leucine zipper deletion, OM-LZΔ) would behave similarly in reprogramming. To verify that AF10 OM-LZΔ mutant is impaired in binding to DOT1L, we performed the DOT1L-BioID assay in the presence of AF10 OM-LZΔ. While WT-AF10 was highly biotinylated by BirA*-DOT1L, we observed minimal biotinylation of the OM-LZΔ mutant (Fig. 3b). In addition, co-immunoprecipitation experiments revealed that HA-tagged DOT1L interacted with WT AF10, but not the AF10 OM-LZΔ mutant (Fig. 3c). The increased reprogramming phenotype upon AF10 suppression was reverted by the L107A but not the OM-LZΔ mutant, indicating that AF10–DOT1L interaction, but not histone binding, is critical for reprogramming (Fig. 3d). L107A mutant AF10 had a negative effect on reprogramming, which was not due to the decreased cell viability (Fig. 3e). However, we observed an aberrant localization pattern of L107A mut AF10 in the nucleus which may interfere with reprogramming (Additional file 1:Fig. S2f). Taken together, these results show that AF10 constitutes a barrier to reprogramming to pluripotency and that its binding to DOT1L is important for this function.

### AF10 expression maintains somatic cell identity

**Fig. 4:**
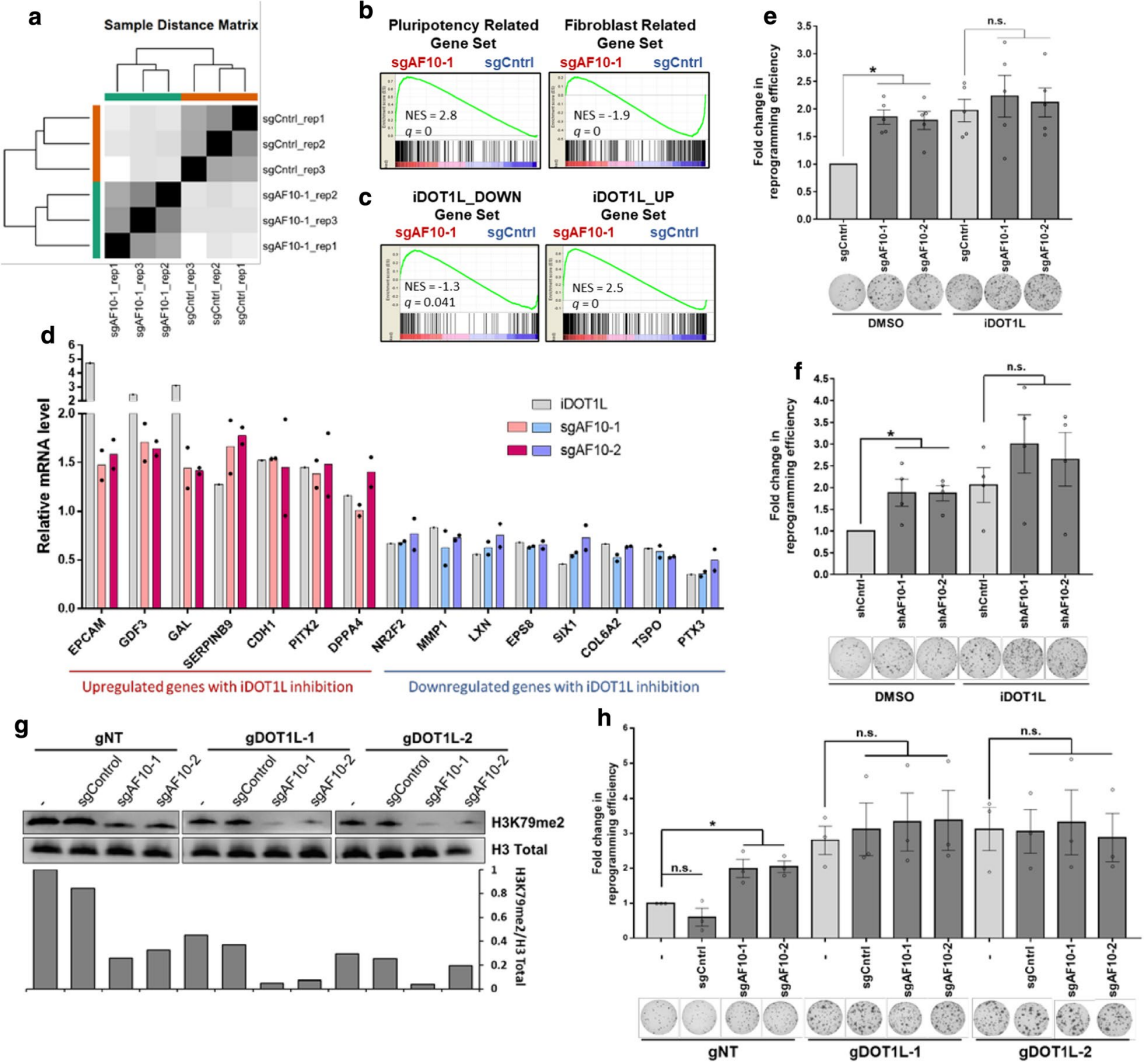
AF10 expression maintains somatic cell identity. **a **Sample distance matrix of RNA-sequencing replicate samples. **b** Gene set enrichment analysis (GSEA) of transcriptome data of sgAF10 cells with respect to pluripotency-related and fibroblast-related gene sets. NES: normalized enrichment score, *q*: false discovery rate (FDR) *q*-value. **c** Gene set enrichment analysis (GSEA) of transcriptome data of sgAF10 cells with respect to iDOT1L_DOWN and iDOT1L_UP gene sets. NES: normalized enrichment score, *q*: false discovery rate (FDR) *q*-value. **d** mRNA levels for a set of DOT1L-regulated genes in AF10 knock-out fibroblasts as determined by qRT-PCR. β-actin was used as an internal control. Gene expression levels were normalized to sgControl expressing fibroblasts for sgAF10 samples and DMSO treated cells for iDOT1L samples. Two biological replicates are indicated for sgAF10 samples and bar graph indicates the average of replicates. **e** Fold change in the number of Tra-1-60-positive colonies derived from AF10 sgRNA expressing cells in the presence of DMSO or a DOT1L inhibitor (iDOT1L; EPZ004777). *P* values were 0.001 for sgAF10-1 and 0.004 for sgAF10-2. n.s. not significant. **f** Fold change in the number of Tra-1-60-positive colonies derived from control or AF10 knock-down cells (shAF10) treated with either vehicle (DMSO) or iDOT1L (EPZ004777). *P* values were 0.034 for shAF10-1 and 0.007 for shAF10-2. n.s., not significant. **g** Immunoblot for H3K79me2 in double KO cells. Total H3 levels were used as loading control. Replicate of this experiment in Additional file 1: Figure S3c. Quantifications were normalized to gNT sample. **h** Fold change in the number of Tra-1-60-positive colonies derived from double knock-out cells expressing DOT1L and AF10 targeting sgRNAs. *P* values were 0.031 for sgAF10-1 and 0.014 for sgAF10-2. n.s. not significant

**Fig. 5:**
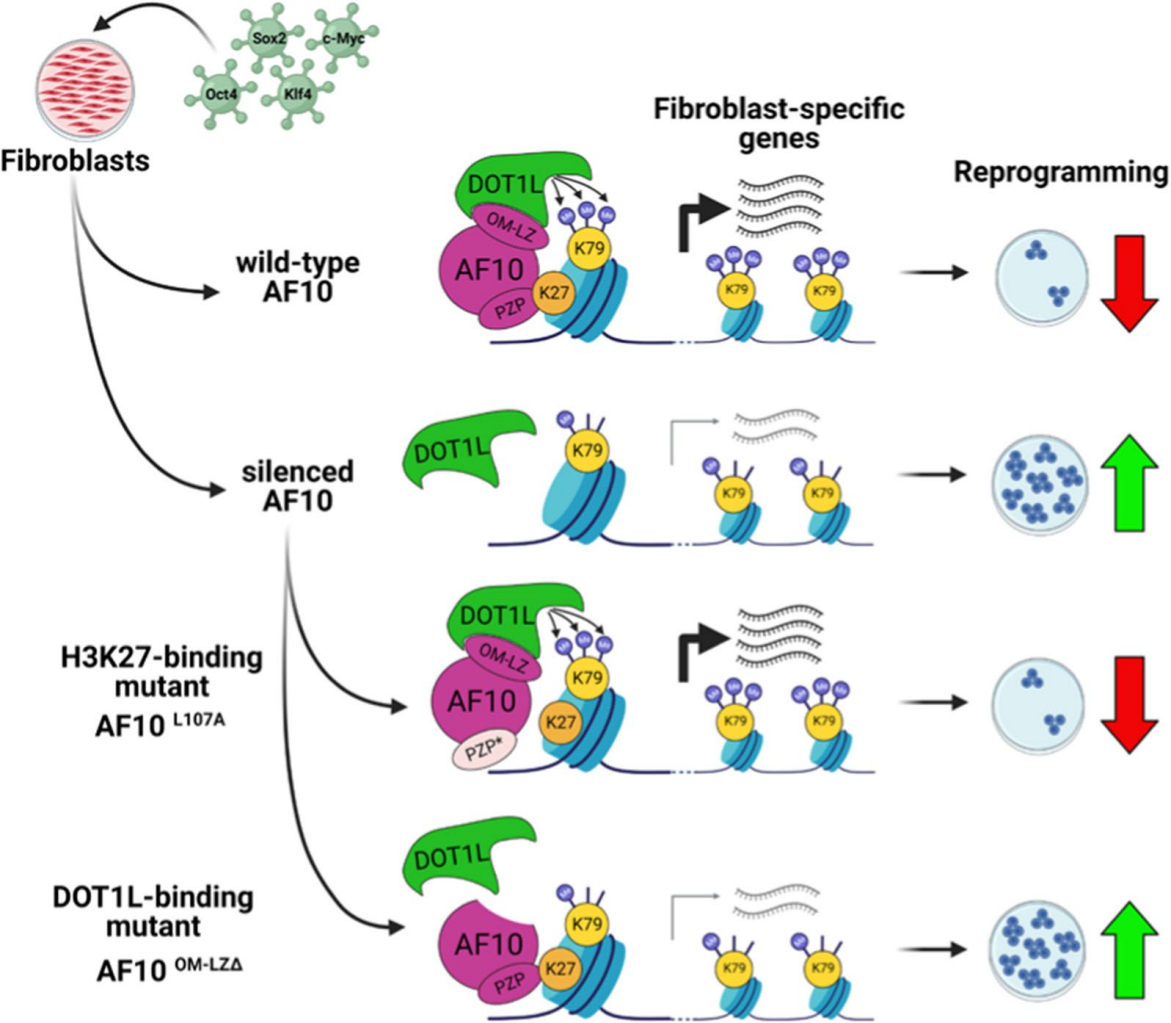
Model for AF10’s role in maintaining somatic cell identity and reprogramming. In the presence of AF10, DOT1L-mediated H3K79 methylation and expression of somatic specific genes are high. Upon silencing of AF10, H3K79me2 is reduced, and somatic specific gene expression is downregulated, resulting in higher reprogramming efficiency. This effect is dependent on AF10–DOT1L interaction but not on AF10 histone binding

To elucidate the mechanism by which AF10 suppression enhances iPSC generation, we investigated the transcriptional changes occurring upon sgAF10 expression. Since AF10 loss has a clear effect of H3K79me2 levels, we hypothesized that it will affect the transcriptional landscape of somatic cells. We performed an RNA-sequencing experiment in sgControl and sgAF10-1 expressing cells early during reprogramming, on day 6 post-OSKM expression. Replicate RNA samples clustered closely, indicating high reproducibility (Fig. 4a). A large number of genes were differentially expressed between control and sgAf10 expressing fibroblasts upon OSKM induction (Additional file 1: Figure S3a). We specifically asked whether pluripotency-associated genes were upregulated upon suppression of AF10. Gene-set enrichment analysis (GSEA) indicated that pluripotency genes were highly enriched in sgAF10 cells upon OSKM expression (Fig. 4b). On the other hand, fibroblast-related genes were negatively enriched upon sgAF10 treatment, which suggested greater suppression of the somatic cell-specific gene expression program (Fig. 4b). We next assessed the degree to which AF10 and DOT1L-induced transcriptional changes overlap during reprogramming. Based on published gene expression data of DOT1L inhibitor-treated cells, we generated gene sets comprising genes negatively or positively regulated by DOT1L [2]. GSEA of sgAF10 transcriptome data revealed that iDOT1L-downregulated genes were negatively enriched, while iDOT1L-upregulated genes were positively enriched upon AF10 loss (Fig. 4c). Several commonly regulated genes such as *EPCAM*, *COL6A2* and *NR2F2* demonstrates similar expression changes in both sgAF10 and iDOT1L samples (Additional file 1: Figure S3b) and this was verified by qPCR (Fig. 4d). Taken together, these data suggest that AF10 suppression and DOT1L inhibition have similar transcriptional effects during reprogramming. We functionally tested this notion by combining AF10 suppression with DOT1L inhibition. Individually, DOT1L inhibition or genetic suppression of AF10 increased reprogramming efficiency as expected; however, the combination of these perturbations did not result in a further increase in efficiency (Fig. 4e, f). We also generated combined knock-out lines of both AF10 and DOT1L, verified the decrease in H3K79 methylation and then reprogrammed the resulting double knock-out cells (Fig. 4g, Additional file 1: Fig. S3c). AF10 and DOT1L double knockout did not significantly increase reprogramming compared to targeting each factor alone (Fig. 4h). Overall, these results indicate that suppression of AF10 increases reprogramming mainly through its effect on DOT1L and H3K79 methylation (Fig. 5).

## Discussion

Here, we identified DOT1L-proximal proteins via proximity labeling and tested the effects of these proteins on somatic cell reprogramming. BioID-based proteomics uncovered TPR, KAISO, NUMA1, MRE11, NONO and SIN3B as novel DOT1L-proximal proteins in addition to known direct interactors of DOT1L, including AF10, AF17, ENL, Histone H1 and DDX21 [11, 22, 25]. We tested the effect of DOT1L-proximal proteins in somatic cell reprogramming via loss of function experiments and showed that AF10 and NONO play functionally important roles in the generation of human iPSCs. Among these proteins, only loss of AF10 affected overall H3K79 methylation levels, prompting us to further investigate its mechanism. AF10 is a member of the Dotcom complex along with AF17, AF9 and ENL [11]. The latter proteins are also present in the Super Elongation Complex (SEC) [26]. The fact that AF9, AF17 and ENL had no effect in reprogramming points to a specific role for AF10 in this process, a finding corroborated in recent studies of mouse reprogramming [27]. This finding also suggests that DOT1L’s role in suppressing cellular reprogramming may be largely independent of its association with transcriptional elongation and its effect on RNA Polymerase II processivity [28]. While SEC activity is required for reprogramming [29], suppression of AF10 may uncouple the function of Dotcom and H3K79 methylation from transcriptional elongation and thus enhance reprogramming.

AF10 is a rate-limiting cofactor for higher order (di- and tri-) methylation of H3K79 and directly interacts with DOT1L through its octamer motif- leucine zipper (OM-LZ) domain [24, 30, 31]. We show that this interaction is critical for AF10’s ability to prevent reprogramming. Furthermore, combined genetic suppression of AF10 and DOT1L did not result in an additive enhancement of reprogramming. Another potential function of AF10 is to act as a histone reader, recognizing unmethylated H3K27 and recruiting DOT1L to loci devoid of H3K27 modifications [24]. However, we find that histone-binding function of AF10 is not necessary to suppress reprogramming. Therefore, AF10 acts as a key barrier to reprogramming not through histone binding, but by regulating higher order H3K79 methylation by DOT1L.

## Conclusions

AF10 suppression in somatic cells results in wide-ranging gene expression changes during reprogramming. In particular, silencing of somatic-specific genes is facilitated by suppression of AF10, a finding in consonance with the effect of DOT1L inhibition. These findings indicate that AF10 acts as a safeguarding mechanism for somatic cell identity by enabling higher order H3K79 methylation of somatic-specific genes. Presence of higher order H3K79 methylation may antagonize gene repression, thereby preventing silencing of somatic transcriptional programs upon OSKM expression [15, 32]. Alternatively, recent work points to a role for DOT1L in transcription initiation, and it will be interesting to investigate if AF10 plays a role in that process [25]. While the role of H3K79 methylation in preventing reprogramming to pluripotency is now well established, it will be of interest to test whether AF10 and DOT1L also regulate direct lineage conversions between terminally differentiated cells.

## Methods

### Plasmids

BirA^R118G^ (BirA*) cDNA was amplified from pcDNA3.1-mycBioID (Addgene, catalog no. 35700). DOT1L wild type (WT) and mutant (G163R/S164C/G165R) cDNAs with HA-tag in pMIY plasmids were described previously [18]. In-frame BirA*-DOT1L fusion protein coding sequence was cloned into pENTR1A no ccDB (Addgene, catalog no. 17398) and transferred into expression plasmid pLEX-307 (Addgene, catalog no. 41392) via LR cloning (Invitrogen). pBabe-puro-AF10 wild-type (wt) and L107A mutant (mut) plasmids were gifts of Or Gozani (Stanford University). Wt- and mut-AF10 cDNAs were amplified with Phusion polymerase and inserted into pENTR1A no ccDB (Addgene, catalog no. 17398). OM-LZ domain (703–784) deleted plasmids were prepared with Q5-site directed mutagenesis kit (NEB) according to manufacturer’s instructions. All AF10 sequences were cloned into a lentiviral expression plasmid pLenti CMV/TO Hygro DEST (Addgene, catalog no. 17291) via LR cloning (Invitrogen). pcDNA5 GFP-AF10 wt and L107A mutant (mut) plasmids were gifts of Or Gozani (Stanford University). OM-LZ domain (703–784) deleted pcDNA5 GFP fusion AF10 OMLZ deleted mutant was prepared with Q5-site directed mutagenesis kit (NEB) according to manufacturer’s instructions.

### shRNA and gRNA cloning

shRNAs were designed and cloned into the MSCV-PM vector as previously described [2]. All vectors were confirmed by Sanger sequencing. sgAF10-1 and 2 plasmids were gifts of Or Gozani (Stanford University). Rest of the gRNAs were designed and cloned into lentiCRISPRv2 (Addgene, catalog no. 52691) vector as previously described [33]. shRNA and sgRNA sequences are listed in Additional file 3: Table S2. All vectors were confirmed by Sanger sequencing using U6 promoter sequencing primer (5′-ACTATCATATGCTTACCGTAAC-3′).

### Reprogramming assays

Fifty thousand dH1f cells [20] were seeded onto 12-well plates and infected with lentiviral OSKM vectors (Addgene, catalog no. 21162, 21164). Medium was changed every other day with D10 medium (1XDMEM with 10% FBS, 1% penicillin/streptomycin). On day 6, cells were trypsinized and transferred onto mitomycin-c treated MEFs. Medium was then changed to hESC medium (DMEM/F12 with 20% KOSR, 1% l-glutamine, 1% non-essential amino acids, 0.055 mM beta-mercaptoethanol, 10 ng ml^−1^ bFGF). Plates were fixed and stained for Tra-1-60 on day 21. iDOT1L (EPZ004777, Tocris) was used at 3 μM concentration for 6 days after OSKM infection.

### Production of viral supernatants

HEK-293T cells were plated at a density of 2.5 × 10^6^ cells per 10-cm dish and transfected with 2.5 µg viral vector, 2.25 µg pUMVC (Addgene, catalog no. 8449) for retroviruses or pCMV-dR8.2 ΔVPR (Addgene, catalog no. 8455) for lentiviruses with 0.25 µg pCMV-VSV-G (Addgene, catalog no. 8454) using 20 µl FUGENE 6 (Promega) in 400 µl DMEM per plate. Supernatants were collected 48 h and 72 h post-transfection and filtered through 0.45-µm pore size filters. To concentrate the viruses, viral supernatants were mixed with PEG8000 (Sigma, dissolved in DPBS, 10% final concentration) and left overnight at 4°C. The next day, supernatants were centrifuged at 2500 rpm for 20 min, and pellets were resuspended in PBS. Viral transductions were carried out overnight in the presence of 8 µg ml^−1^ protamine sulfate (Sigma). Transduced cells were selected with 1 μg ml^−1^ puromycin or 200 μg ml^−1^ hygromycin.

### Generation of DOT1L-KO single-cell clones

HEK293T cells were transfected with either non-targeting (gControl) or guideRNA DOT1L (gDOT1L) containing lenticrisprV2 plasmids and transfected cells were selected with 2 μg ml^−1^ puromycin. After selection, cells were trypsinized, diluted to a single-cell suspension and seeded onto 96-well plates. Single cell clones were identified and expanded. H3K79me2 levels in selected single-cell clones were assayed via immunoblotting. sgControl clone #1 and sgAF10-1 clone #1 were used for teratoma formation assay and  sgAF10-2 clone #1 immunofluorescence experiments.

### Quantitative RT-PCR analyses

Total RNA was extracted using NucleoSpin RNA kit (Macherey Nagel) and reverse transcribed with Hexanucleotide Mix (Roche). The resulting complementary DNAs were used for PCR using SYBR-Green Master PCR mix (Roche) and run on a LightCycler 480 Instrument II (Roche) with 40 cycles of 10 s at 95 °C, 30 s at 60 °C and 30 s at 72 °C. All quantifications were normalized to an endogenous β-actin control. The relative quantification value for each target gene compared to the calibrator for that target is expressed as 2^−(Ct − Cc)^ (Ct and Cc are the mean threshold cycle differences after normalizing to β-actin). List of primers are in Additional file 3: Table S2.

### RNA sequencing and analysis

RNA isolation was performed with Direct-zol kit (Zymo Research). NEBNext Poly(A) mRNA Magnetic Isolation Module from NEBNext Ultra Directional RNA Library Prep Kit for Illumina was used to enrich mRNA from RNA-sequencing samples. Samples were then validated on a Tapestation (Agilent) to determine library size and quantification prior to paired-end (2 × 41 bp) sequencing on a NextSeq 500 (Illumina) platform. Reads were mapped to hg19 built-in genome by HISAT2 after assessing their quality by FastQC. RNA-sequencing data are deposited to the NCBI GEO database with the accession number GSE161043. DeSeq2 package was used to find differentially expressed genes between samples. Genes were considered as differentially regulated based on |log2 fold change|> 0.5 and adjusted *p*-value < 0.05. Differential gene expressions between pluripotent stem cells and fibroblast cells were computed by affy and limma packages from R to generate fibroblast- and pluripotency-related gene sets as described previously [6]. Differential gene expression analysis to generate iDOT1L regulated gene sets is performed on GEO2R web tool between dH1f-inhibitor-OSKM samples and dH1f-untreated-OSKM samples from GSE29253 [2]. iDOT1L_UP gene set is composed of genes that are upregulated in treatment group (*p*-value < 0.05 and logFC > 0.5) and iDOT1L_DOWN gene set is composed of genes that are downregulated in treatment group (*p*-value < 0.05 and logFC < − 0.5). Rank-ordered gene lists were used for gene-set enrichment analysis [34].

### Nuclear protein extraction and histone acid extraction

Cell pellets were resuspended in cytosolic lysis buffer (10 mM HEPES pH7.9, 10 mM KCl, 0.1 mM EDTA, 0.4% NP-40, cOmplete ULTRA protease inhibitor Tablets [Roche]) and incubated for 15 min on ice and centrifuged at 4 °C for 3 min at 3000* g*. Pellets were washed once with cytosolic lysis buffer and then resuspended in nuclear lysis buffer (20 mM HEPES pH7.9, 0.4 M NaCl, 1 mM EDTA, 10% glycerol, cOmplete ULTRA protease inhibitor Tablets [Roche]) followed by sonication 2 times for 10 s at 40 amplitude with a 10 s interval in between (QSONICA Q700 with microtip). After sonication, tubes were centrifuged at 4 °C for 5 min at 15000*g*. Supernatant was removed as nuclear protein fraction. For histone acid extraction, cell pellets were resuspended with Triton extraction buffer (0.5% Triton X-100, 2 mM PMSF, 0.02% NaN_3_ in PBS) and incubated for 10 min on ice then centrifuged at 4 °C for 10 min at 2000 rpm. Pellet was washed with triton extraction buffer and centrifuged again. Supernatant was discarded and the pellet was resuspended in 0.2 N HCl. Tubes were incubated at 4 °C for 16 h on a rotating wheel and centrifuged at 4 °C for 10 min at 2000 rpm. Supernatants were neutralized with the addition 0.1 M NaOH for 1/5 volume of HCl solution. Protein concentrations were determined via BCA assay (Thermo Scientific).

### Immunoblotting

Equal amounts of proteins were boiled with loading buffer (4 × Laemmli sample buffer, Bio-Rad) and loaded onto 4–15% Mini-PROTEAN TGX Precast Protein Gels (Bio-Rad). Gels were run with TGS buffer (diluted from 10 × stock, Bio-Rad). Precision Plus Protein Dual Color Standards (Bio-Rad) were used a molecular weight ladder. Proteins were transferred onto Immun-Blot PVDF Membrane (Bio-Rad) via Trans-Blot Turbo Transfer System (Bio-Rad). Membrane was incubated with 5% blotting grade blocker (Bio-Rad) dissolved in TBS-T (20 mM Tris, 150 mM NaCl, 0.1% Tween 20 – pH 7.6). For Streptavidin-HRP blotting membranes were blocked with 2% bovine serum albumin (BSA, Sigma) in TBS-T. Primary antibodies were incubated on membranes at 4 °C for 16 h. Primary antibodies were Streptavidin-HRP (BioLegend 405,210, 1:10,000), H3K79me2 (ab3594, 1:1000), H3 total (ab1791, 1:1000 in Additional file 1: Figure S1b), H3 total (CST4499, 1:1000 rest of the H3 blots). After primary antibody incubation, membranes were washed and then incubated with secondary antibody solution (1:5000 secondary antibody ab97051 in 5% blotting grade blocker in TBS-T) at room temperature for 1–2 h. Membranes were washed with TBS-T and proteins were visualized with Pierce ECL Western Blotting Substrate (Thermo Scientific) and Odyssey Fc Imaging systems (LiCor). Quantifications were performed via LiCOR.

### Pull-down assays and mass spectrometry analysis for BioID

HEK-293T cells were infected with lentiviral BirA*-DOT1L. Puromycin selected cells were expanded and incubated with 50 μM D-Biotin (Sigma, 47868) for 24 h. Proteins were obtained via nuclear fractionation method. As a control, uninfected HEK293T cells were used. Pull-down was performed with Streptavidin beads (Thermo Scientific, 53117) as previously described [35]. Briefly, 3 mg nuclear fraction was incubated with 100 μl Streptavidin beads at 4 °C for 16 h on a rotating wheel at 10 rpm. Then supernatants were collected, and beads were washed twice in 2% SDS; once with wash buffer 1 (0.2% deoxycholate, 1% Triton X, 500 mM NaCI, 1 mM EDTA, 50 mM HEPES, pH 7.5), once with wash buffer 2 (250 mM LiCI, 0.5% NP-40, 0.5% deoxycholate, 1% Triton X, 500 mM NaCI, 1 mM EDTA, 10 mM Tris, pH 8.1) and twice with wash buffer 3 (50 mM Tris, pH 7.4, and 50 mM NaCI). Eluted proteins were analyzed with AF10 (sc27083) antibody to observe the efficiency of pull-down. For mass spectrometry analysis, control (uninfected) and BirA*-AF10 WT or MUT expressing HEK293T cells were used. Following nuclear protein isolation and streptavidin pulldown, bound proteins were digested with on-bead tryptic proteolysis as previously described [36]. Briefly, beads were washed (8 M urea in 0.1 M Tris–HCl, pH 8.5) and reduction and alkylation steps performed. After a final wash with 50 mM ammonium bicarbonate, beads were treated with trypsin overnight. Reaction was quenched with acidification and the resulting peptides were desalted [37] and then analyzed with reversed-phase nLC (NanoLC-II, Thermo Scientific) combined with orbitrap mass spectrometer (Q Exactive Orbitrap, Thermo Scientific). The raw files were processed with Proteome Discoverer 1.4 (Thermo Scientific) using human Uniprot database (Release 2015–21,039 entries) as previously described [36, 38]. Two technical replicates were performed for each sample. Raw data from LC–MS/MS analysis can be found in Additional file 4: Table S3. To identify DOT1L-specific biotinylation, proteins detected in HEK293T control samples were subtracted from BirA* infected samples. The remaining proteins were selected only if were present in both runs of mass spectrometry. Among these common proteins, nuclear localized ones are determined via GO annotation (http://www.geneontology.org/) using cellular component analysis. UniProt protein names were converted via ID mapping tool (https://www.uniprot.org/uploadlists/). Determined proteins were sorted according to their sequence coverage and abundance using PSM (peptide spectrum matches) numbers. CRAPome analysis was performed via CRAPOME2.0 (https://reprint-apms.org/?q=chooseworkflow) [19].

### Co-immunoprecipitation assay

HEK-293T cells were plated at a density of 5 × 10^6^ cells per 15-cm dish and transfected with 10 µg DOT1L-HA and AF10 vectors using FUGENE 6 (Promega). After 48 h, cells were harvested and lysed with Pierce IP-Lysis Buffer (ThermoScientific) with cOmplete ULTRA protease inhibitor Tablets (Roche). Lysates were incubated with IgG and HA antibodies at 4 °C for 16 h on a rotating wheel at 10 rpm. Pre-washed DynaBead Protein A (Thermo Fisher) were added and incubated at 4 °C for 4 h. Beads were washed with lysis buffer for 3 times with 10 min intervals. Beads were boiled for 10 min in 4X Laemmli sample buffer (Bio-Rad). Half of the eluted proteins were loaded in gels and immunoblotted with AF10 antibody (sc27083). 1/15 of input samples were immunoblotted with AF10 (sc27083), DOT1L (A300-953A, Bethyl Laboratories) and Actin (ab8227) antibodies.

### Tra-1-60 staining and quantification

To quantify the number of iPSC colonies, reprogramming plates were stained with Tra-1-60 antibody as previously described [2]. Briefly, cells were fixed with 4% paraformaldehyde and incubated with biotin-anti-Tra-1-60 (BioLegend, catalog no. 330604, 1:250) diluted in PBS with 3% FBS and 0.3% Triton X-100. Followed by incubation with streptavidin-HRP (Biolegend, catalog no. 405210, 1:500). Staining was developed with the DAB peroxidase substrate solution (0.05% 3,3′-diaminobenzidine [Sigma, D8001], 0.05% nickel ammonium sulfate and 0.015% H_2_O_2_ in PBS, pH 7.2) and iPSC colonies were quantified with ImageJ software (https://imagej.nih.gov/ij/).

### T7-endonuclease assay

gRNA infected cells were harvested, and genomic DNAs were isolated using MN Nucleospin Tissue kit. gRNA targeting sites were amplified with specific primers (Additional file 3: Table S2) PCR clean-up was performed (MN, PCR clean up and gel extraction kit). 400 ng from cleaned PCR products were mixed with NEB 2 buffer and incubated according to heteroduplex formation protocol (5 min at 95 °C and ramp down to 85 °C at − 2 °C/s and ramp down to 25 °C at − 0.1 °C/s). After heteroduplex formation, samples were treated with T7 endonuclease (NEB) for 1–2 h at 37 °C. Digested samples were analyzed on 2% agarose gels and visualized via Gel Doc XR System (Bio-Rad).

### Teratoma formation assay

All experiments were carried out under a protocol approved by Koç University Animal Experiments Ethics Committee. Injections were performed as previously described [39]. Briefly, iPSCs from 80% confluent 10 cm dish were collected using ReLeSR (Stemcell Technologies) and resuspended in 100 μl ice-cold 1:1 mixture of Matrigel (Corning) and hES growth medium. Intramuscular injections were performed in SCID mice. Teratomas were collected 8–10 weeks after injection and analyzed histologically via hematoxylin and eosin staining.

### Immunofluorescence staining

Immunostainings were performed as previously described [6]. Briefly, iPSCs from single-cell clones were fixed with 4% paraformaldehyde in PBS and incubated overnight at 4 °C with primary antibody: OCT4, (Abcam, ab19857), SSEA4 (BD, 560219), NANOG (Abcam, ab21624). Nuclei were stained with DAPI (Vectashield, H-1500). Images were acquired using a Nikon 90i confocal microscope.

### Cell viability assay

AF10 plasmids transduced with dH1f cells and selected with Hygromycin. After 10 days, cells were seeded in black 96-well plates as 5000 cells/well in triplicates. Cell viability was detected with Cell Titer Glo assay (Promega) according to manufacturer’s instructions.

## Supplementary Information

**Additional file 1: Figure S1.** Identification of proximal interactors of DOT1L via BioID and their effect on reprogramming. (a) Replicate immunoblot of Fig. 1b. (b) CRAPome analysis of WT-DOT1L proximal proteins identified via BioID assay. Y-axis shows the PSM values from MS data. (c) mRNA levels of shRNA targeted genes were assessed via qRT-PCR. β-actin was used as an internal control and gene expression levels are normalized to control shFF (firefly luciferase targeting shRNA) expressing cells. (d) mRNA levels of shAF9 targeted genes were assessed via qRT-PCR. β-actin was used as an internal control and gene expression levels are normalized to control shFF (firefly luciferase targeting shRNA) expressing cells. (e) Fold change in the number of Tra-1-60 positive colonies upon shAF9 expression. *P* values were determined by one sample t-test; * *P* < 0.05. Bar graphs show the mean and error bars represent SEM in three independent biological replicates. Representative Tra-1-60 stained wells are shown below the graph. *P* values were 0.01 for shAF9-1 and 0.1 for shAF9-2. (f) Immunoblot for H3K79me2 in shRNA-targeted fibroblasts. Total H3 levels were used as loading control. **Figure S2**. Validation of AF10 inhibition in somatic cells and iPSCs. (a) T7-endonuclease assay for sgAF10 target sites (top). Expected DNA fragments are indicated with white arrow heads. (b) AF10 mRNA levels in control and sgAF10 expressing cells as determined by qRT-PCR. β-actin was used as an internal control and expression level is normalized to sgControl expressing cells. qRT-PCR primer binding sites are depicted on the top panel. (c) Replicate immunoblot of Fig. 2b. (d) AF10 mRNA levels in individual iPSC clones derived from control and AF10 sgRNA expressing fibroblasts as determined by qRT-PCR. β-actin was used as an internal control and expression level is normalized to sgControl-1 iPSCs. (e) Replicate immunoblot of Fig. 3d. (f) Confocal images of HEK-293T transfected with GFP-AF10-WT, GFP-AF10-L107A and GFP-AF10-OM-LZ∆ expressing plasmids. Scale bar represents 10 μm. DAPI shows nuclear staining. **Figure S3**: AF10 expression maintains somatic cell identity similar to DOT1L. (a) Number of differentially expressed genes for sgAF10-1 RNA sequencing and iDOT1L samples. (b) Fold change differences of selected genes that are regulated with DOT1L in RNA sequencing samples of sgAF10-1 and iDOT1L. (c) Replicate immunoblot of Fig. 4g.

**Additional file 2: Table S1**. List of proximal interactors of DOT1L-WT and DOTL1-MUT and CRAPome analysis.

**Additional file 3 Table S2**. List of oligonucleotides for cloning and PCR.

**Additional file 4 Table S3**. Raw data of mass spectrometry analysis of BioID.

## Data Availability

RNA-sequencing data are deposited to the NCBI GEO database with the accession number GSE161043.
