# Supplementary material for: AF10 (MLLT10) prevents somatic cell reprogramming through regulation of DOT1L-mediated H3K79 methylation
Source: Epigenetics Chromatin. 2021 Jul 2;14:32. doi: 10.1186/s13072-021-00406-7 (PMC8254283; doi:10.1186/s13072-021-00406-7)
Supplement: Supplementary file 1 — Additional file 1: Figure S1. Identification of proximal interactors of DOT1L via BioID and their effect on reprogramming. (a) Replicate immunoblot of Fig. 1b. (b) CRAPome analysis of WT-DOT1L proximal proteins identified via BioID assay. Y-axis shows the PSM values from MS data. (c) mRNA levels of shRNA targeted genes were assessed via qRT-PCR. β-actin was used as an internal control and gene expression levels are normalized to control shFF (firefly luciferase targeting shRNA) expressing cells. (d) mRNA levels of shAF9 targeted genes were assessed via qRT-PCR. β-actin was used as an internal control and gene expression levels are normalized to control shFF (firefly luciferase targeting shRNA) expressing cells. (e) Fold change in the number of Tra-1-60 positive colonies upon shAF9 expression. P values were determined by one sample t-test; * P < 0.05. Bar graphs show the mean and error bars represent SEM in three independent biological replicates. Representative Tra-1-60 stained wells are shown below the graph. P values were 0.01 for shAF9-1 and 0.1 for shAF9-2. (f) Immunoblot for H3K79me2 in shRNA-targeted fibroblasts. Total H3 levels were used as loading control. Figure S2. Validation of AF10 inhibition in somatic cells and iPSCs. (a) T7-endonuclease assay for sgAF10 target sites (top). Expected DNA fragments are indicated with white arrow heads. (b) AF10 mRNA levels in control and sgAF10 expressing cells as determined by qRT-PCR. β-actin was used as an internal control and expression level is normalized to sgControl expressing cells. qRT-PCR primer binding sites are depicted on the top panel. (c) Replicate immunoblot of Fig. 2b. (d) AF10 mRNA levels in individual iPSC clones derived from control and AF10 sgRNA expressing fibroblasts as determined by qRT-PCR. β-actin was used as an internal control and expression level is normalized to sgControl-1 iPSCs. (e) Replicate immunoblot of Fig. 3d. (f) Confocal images of HEK-293T transfected with GFP-AF10-WT, GFP-AF10-L107 [file 13072_2021_406_MOESM1_ESM.pptx]

## Slide 1
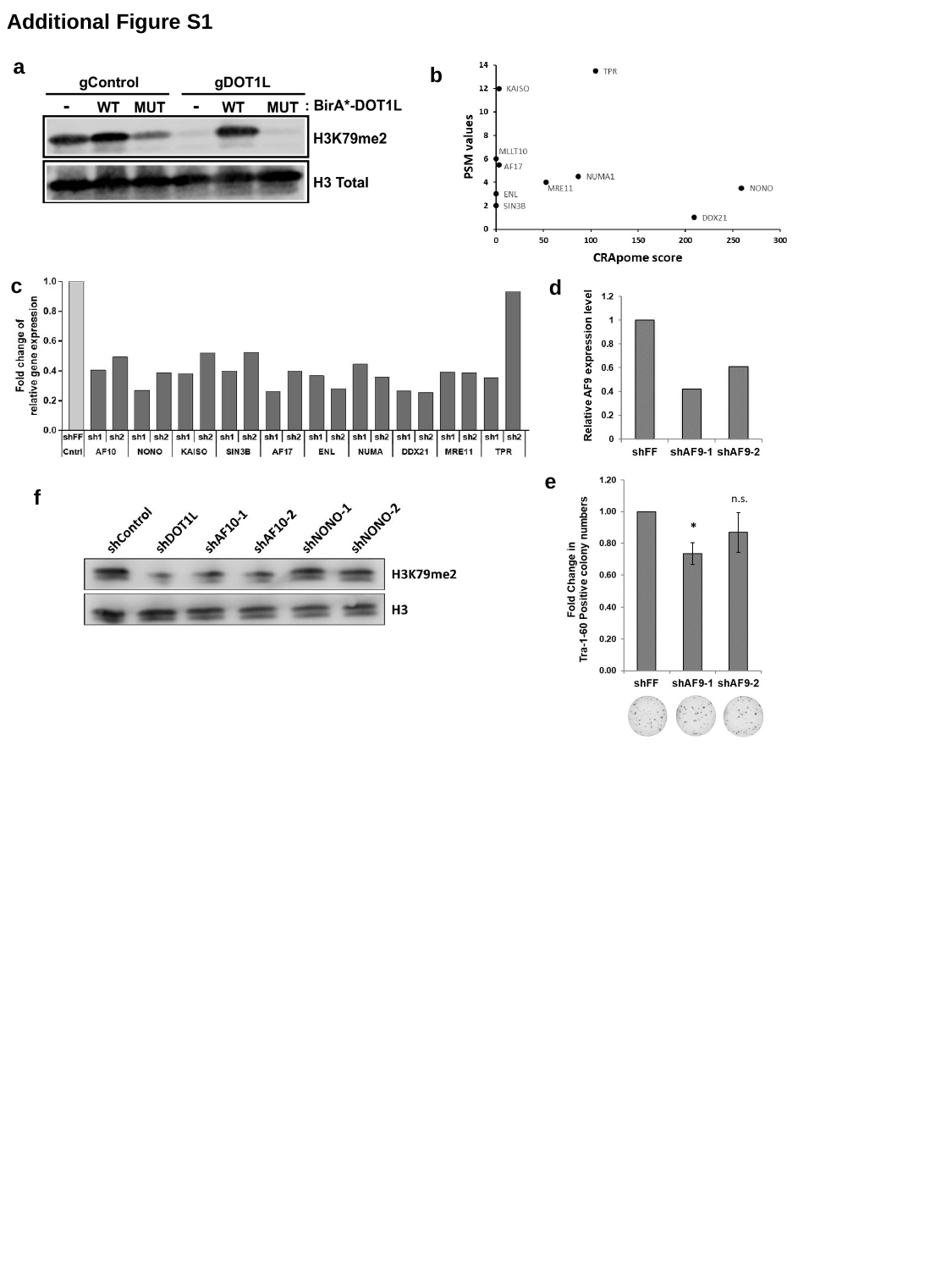

Additional Figure S1
a
b
c
d
e
f

## Slide 2
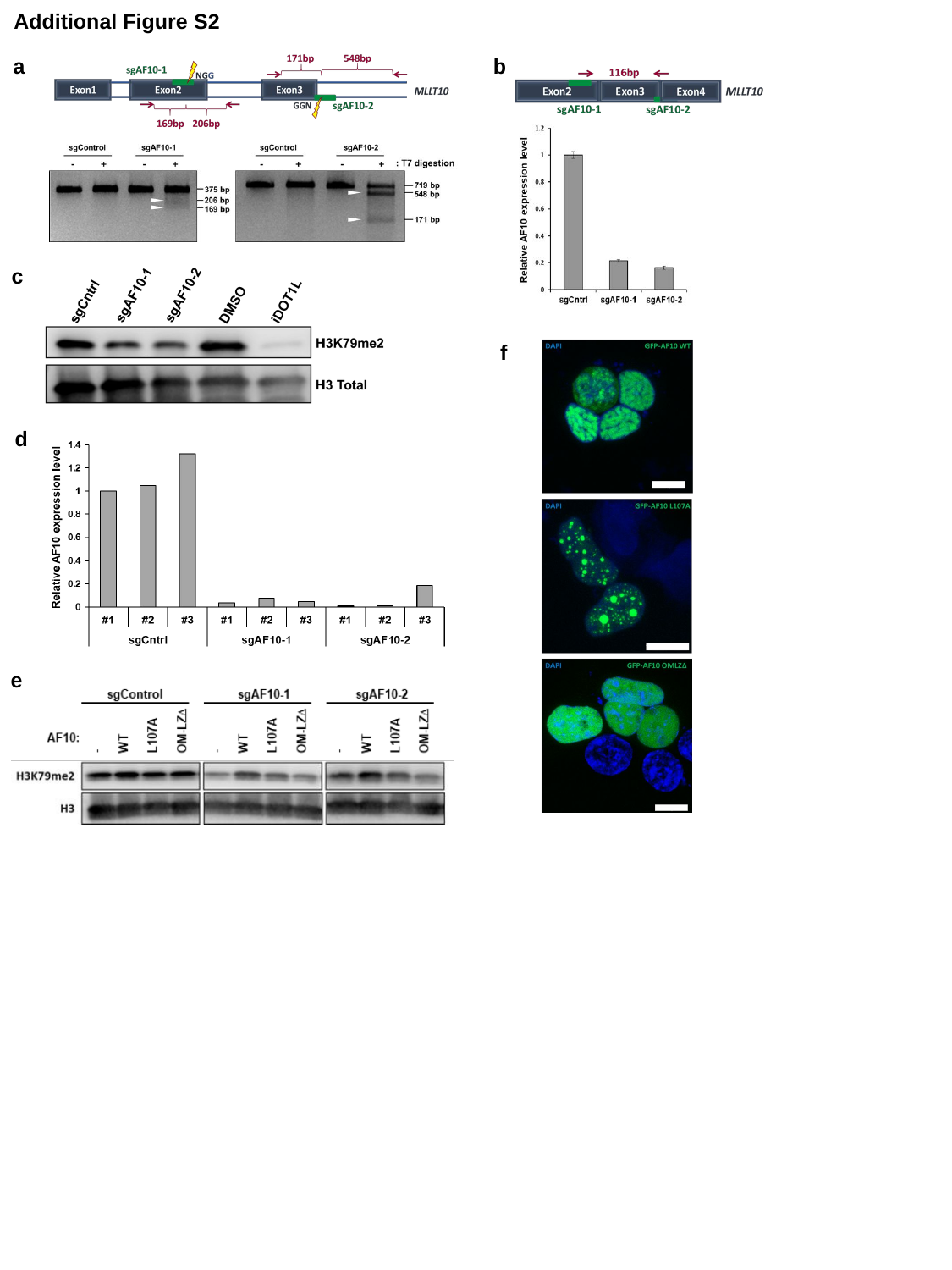

Additional Figure S2
b
a
c
f
d
e

## Slide 3
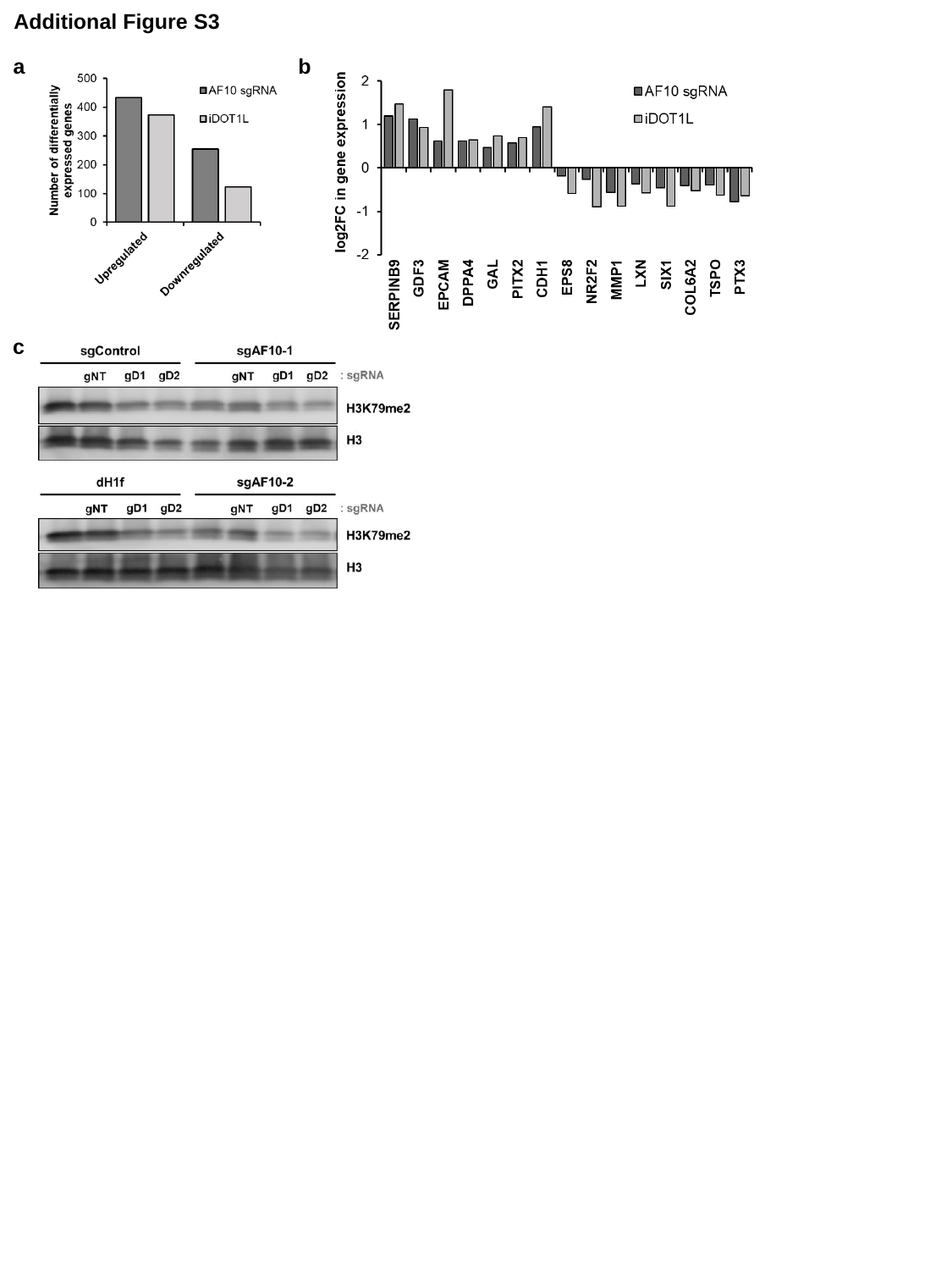

Additional Figure S3
b
a
c
